# Advances in porcine stem cell research and their applications in agriculture

**DOI:** 10.3389/fcell.2026.1862107

**Published:** 2026-06-29

**Authors:** Yangli Pei, Rong Zhou

**Affiliations:** 1 Guangdong Provincial Key Laboratory of Animal Molecular Design and Precise Breeding, Key Laboratory of Animal Molecular Design and Precise Breeding of Guangdong Higher Education Institutes, School of Animal Science and Technology, Foshan University, Foshan, Guangdong, China; 2 The State Key Laboratory of Animal Biotech Biotech Breeding, Institute of Animal Science, Chinese Academy of Agricultural Sciences, Beijing, China; 3 College of Agriculture and Biology, Liaocheng University, Liaocheng, China

**Keywords:** breeding, cultured meat, embryonic stem cells, induced pluripotent stem cells, porcine

## Abstract

Porcine stem cell research has advanced considerably over the past decade, driven by the dual promise of biomedical modeling and agricultural innovation. This review provides a comprehensive synthesis of recent progress in the isolation, characterization, and directed differentiation of major porcine stem cell types, including embryonic stem cells (pESCs), induced pluripotent stem cells (piPSCs), germline stem cells (pGSCs, spermatogonial stem cells and the controversially debated female germline stem cells), and various adult stem cell populations (e.g., muscle satellite cells, adipose-derived stem cells). We critically analyze persistent challenges that hinder translational applications. For pESCs and piPSCs, achieving stable, germline-competent pluripotency remains challenging. This difficulty arises from species-specific epigenetic roadblocks, such as aberrant DNA methylation and incomplete X-chromosome reactivation, as well as suboptimal culture conditions. For pGSCs, the lack of validated molecular markers for spermatogonial stem cells persists, and the existence of female germline stem cells in postnatal ovaries remains scientifically debated. Focusing on agricultural translation, we examine two key application domains. First, in cultured meat production, porcine muscle and adipose stem cells (or pluripotent stem cell-derived progenitors) are cultivated *in vitro* to generate muscle and fat tissues. While this approach offers a promising complement to conventional livestock farming, major hurdles remain, including loss of stemness during large-scale expansion, the high cost of serum-free media, the need for edible scaffolds that replicate meat texture, and regulatory approval pathways. Second, in stem cell-based genetic breeding, concepts such as *in vitro* breeding (combining genomic selection with *in vitro* gametogenesis) could dramatically accelerate genetic gain. However, realization requires robust, species-optimized protocols for complete *in vitro* gametogenesis in pigs, which have not yet been achieved. Finally, we integrate ethical and regulatory considerations, emphasizing that proactive frameworks must evolve in parallel with scientific advances to ensure responsible innovation. By highlighting both the transformative potential and the remaining technical, biological, and regulatory barriers, this review aims to guide future research toward sustainable, efficient, and welfare-conscious swine agriculture.

## 1 Introduction

Stem cell technologies have opened transformative opportunities in both biomedical research and agriculture. Notably, the pig (*Sus scrofa*) has emerged as a key large-animal model due to its physiological similarity to humans and its immense agricultural value (Do et al., 2013). Researchers are actively investigating diverse porcine stem cell types, including porcine embryonic stem cells (pESCs), porcine induced pluripotent stem cells (piPSCs), porcine germline stem cells (pGSCs), and diverse adult stem cell populations (Xu et al., 2019; Xiang et al., 2024; Wang et al., 2023; Vincent et al., 2025). Within the pGSC domain, it is important to note that the existence and functionality of female germline stem cells (FGSCs) in adult mammalian ovaries remains a subject of considerable debate. While some studies support their isolation, strong opposing views and lineage-tracing suggest a lack of consensus regarding their regenerative capacity *in vivo*. Overall, investigations into porcine stem cells aim to address fundamental questions in mammalian pluripotency and to seamlessly translate bench discoveries into industrial applications. These applications include precision genome engineering, advanced reproductive technologies [such as *in vitro* breeding and genomic selection, as described by Goszczynski et al. (2019)], tissue engineering, disease modeling, xenotransplantation, and cellular agriculture.

Over the past decade, substantial advances have been made in deriving pESCs from multiple embryonic sources, optimizing culture conditions, and enhancing piPSC reprogramming protocols (Xiang et al., 2024; Choi H. et al., 2024; Reddy Yeddula et al., 2024). However, achieving a stable pluripotent state capable of long-term maintenance without epigenetic instability, differentiation bias, or loss of germline chimerism remains a central challenge. Germline transmission is a crucial capability for the transgenerational propagation of genetic traits and serves as the gold standard for authentic pluripotency (Samruan et al., 2025). The difficulty in achieving this is not merely a technical hurdle but stems from profound biological barriers. Specifically, pESCs struggle with germline competence due to severe epigenetic roadblocks, such as aberrant DNA methylation, incomplete X-chromosome reactivation, and lingering somatic memory in cloned embryos. Furthermore, traditional culture conditions often fail to adequately support the naïve ground-state epigenetic landscape required for germ cell specification, a state that has been extensively characterized in mouse and human models but remains notoriously difficult to capture and stabilize in ungulate species.

This review synthesizes recent advances within an integrated framework. We critically analyze the derivation of pESCs and piPSCs, highlighting the porcine-specific pluripotency network and comparing it with established murine and human systems. We subsequently bridge these cellular technologies to agricultural applications, providing critical insights into cultured meat production (e.g., scalability and cost comparisons of serum-free media) and the economics of stem cell-based breeding. Finally, we frame these advancements within the necessary ethical and regulatory pathways (such as FDA and EFSA approval processes for cultured meat and gene-edited livestock) to provide a balanced and practical perspective for researchers and industry stakeholders alike.

## 2 Methods

### 2.1 Literature search strategy

A systematic literature search was conducted in PubMed and Web of Science (WoS) databases from their inception to April 2026. The search strategy utilized Boolean operators to combine terms related to four primary domains: porcine embryonic stem cells (pESCs), porcine induced pluripotent stem cells (piPSCs), porcine germline stem cells (pGSCs), and cultured meat. Detailed database-specific search strings and the initial number of records retrieved for each topic are provided in Supplementary Table S1.

### 2.2 Literature screening and selection criteria

In our literature screening and selection process, a multi-tiered set of criteria was applied. All records were imported into EndNote software for deduplication, followed by title and abstract screening by two independent reviewers based on predefined eligibility criteria. General exclusion criteria included non-English publications, conference abstracts lacking full texts, and studies where porcine models were only tangentially discussed.

Specific inclusion and exclusion criteria were customized for each research domain. Ultimately, we selected a range of relevant studies across the fields of pESCs, piPSCs, pGSCs, and cultured meat. To avoid redundancy with a recent comprehensive review detailing derivation methodologies up to early 2024 (Neira et al., 2024), we employed a targeted two-pronged selection strategy for piPSCs. This approach ensured that our study complemented existing literature while focusing on novel and landmark studies. To ensure methodological transparency and reproducibility, the entire literature selection process is visualized in Supplementary Figure S1. A detailed account of the screening process and outcomes is provided in the Supplementary Table S1.

Furthermore, because relevant breeding applications are frequently discussed within broader pESC/piPSC research, data concerning this topic were extracted directly from the included stem cell literature rather than through an independent search, thereby ensuring thematic consistency and preventing redundancy.

## 3 Porcine embryonic stem cells

Significant progress has been made in pESC research since the early 1990s. Researchers have successfully derived and characterized pESCs from various sources, including *in vivo* matured embryos, *in vitro* fertilized (IVF) embryos, parthenogenetic (PA) embryos, and somatic cell nuclear transfer (SCNT) embryos. However, despite the isolation of numerous cell lines that share features with *bona fide* embryonic stem cells (ESCs), the field has consistently been hampered by the inability to generate germline-competent pESCs, which remains the gold-standard criterion for authentic pluripotency in mouse models (Choi et al., 2019; Gao et al., 2019).

Historically, pESC derivation progressed from basic feeder-dependent systems yielding ES-like cells (Piedrahita et al., 1990a; 1990b; Wianny et al., 1997) to defined conditions supporting trilineage differentiation, albeit with limited chimeric integration (Vassiliev et al., 2011; Tan et al., 2012). Recently, the field has advanced significantly through the use of sophisticated inhibitor cocktails (e.g., targeting GSK3β, SRC, and MEK pathways) to derive pESCs with enhanced pluripotency (Gao et al., 2019; Zhi et al., 2022). These efforts have culminated in the establishment of porcine expanded potential stem cells (EPSCs) and chemically defined pESCs. These recent lines exhibit transcriptomic profiles resembling the early embryonic epiblast and possess broad developmental potency, including the capacity to generate both embryonic and extraembryonic lineages (Choi H. et al., 2024; Xiang et al., 2024).

Despite these limitations, a critical conceptual challenge remains: the classification of the pluripotent state captured in pig cells. In mice and humans, a clear distinction exists between the naïve (inner cell mass-like) and primed (epiblast-like) pluripotent states. In pigs, the majority of reported pESC lines appear to be locked in a primed state, evidenced by their flattened colony morphology, dependence on FGF/Activin signaling, and inability to efficiently contribute to chimeras (Choi et al., 2019; Zhang et al., 2015). Establishing a stable, long-term naïve-like state in pigs has proven exceptionally difficult. This barrier is likely due to species-specific differences in the core pluripotency circuitry, such as the delayed expression of key transcription factors like NANOG during porcine blastocyst development compared to mice (Hall and Hyttel, 2014; Chai et al., 2024).

Furthermore, attempts to convert primed pESCs to a naïve state using small-molecule inhibitors have yielded inconsistent responses across laboratories. This highlights that the signaling networks governing pluripotency in pigs are not simply a replica of those in mice or humans. For example, Wnt pathway manipulation has produced contradictory outcomes: while Wnt inhibition (e.g., via IWR-1 or XAV939) can safeguard pluripotency by preventing extraembryonic endoderm differentiation (Wang et al., 2025) reports indicate that simultaneous Wnt activation and inhibition are required to enhance cell viability (Li et al., 2020). Such discrepancies underscore the context-dependent nature of pathway regulation in porcine cells.

Translational applications of pESCs in agriculture, such as *in vitro* breeding, precision gene editing for disease resistance, and cultured meat, hold significant promise. However, their practical implementation will require overcoming unresolved questions regarding long-term epigenetic stability (e.g., progressive erosion of X-chromosome inactivation and abnormal imprinting patterns), differentiation bias under large-scale culture, and the establishment of clear regulatory frameworks.

### 3.1 Different embryonic origins in porcine embryonic stem cells isolation

**FIGURE 1:**
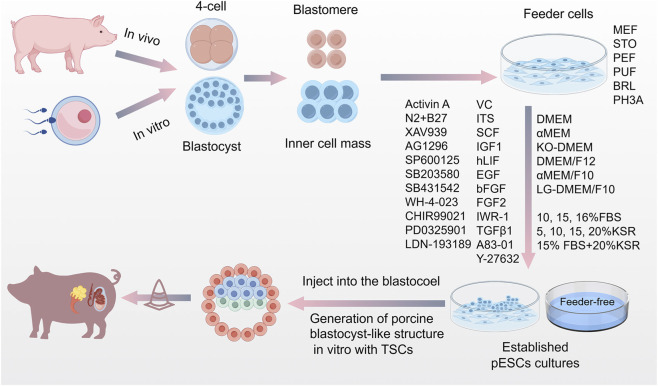
Porcine embryonic stem cell isolation process. Blastomeres or inner cell masses are isolated from embryos at the 4-cell stage to the blastocyst stage, which are either *in vivo* matured or *in vitro* matured. The isolated cells are cultured on feeder cells for embryonic stem cell line establishment. The isolation and culture systems vary among different laboratories. After cell line establishment, the cells can be continuously cultured on feeder cells or in a feeder-free system. These cells can be injected into blastocysts to achieve embryonic chimerism, or the isolated ES cells can be directly used to differentiate into blastocyst-like structures *in vitro* together with trophoblast stem cells (TSCs). However, germline chimeric pigs have not been obtained so far. Note: hLIF: Human Leukemia Inhibitory Factor; bFGF: Basic Fibroblast Growth Factor; EGF: Epidermal Growth Factor; ITS: Insulin-transferrin-Selenium; Y-27632: Rho-associated protein kinase inhibitor; CHIR99021: GSK-3β inhibitor; PD0325901: ERK1/2 inhibitor; VC: Vitamin C; IGF1: Insulin-like Growth Factor 1; TGF β1: Transforming Growth Factor-β1; FGF2: Fibroblast Growth Factor 2; SP600125: JNKi; SB203580: P38i; IWR-1: Wnt inhibitor; WH-4-023: Lck/Src inhibitor; XAV939: Tankyrase inhibitor; AG1296: Tyrosine kinase inhibitor; SCF: Stem Cell Factor; N2: N2 Supplement; B27: B27 Supplement; SB431542: TGF-β pathway inhibitor, A83-01: inhibitor of ALK5 (activin receptor-like kinase 5); LDN-193189: BMP (bone morphogenetic protein) signaling pathway inhibitor; DMEM: Dulbecco’s Modified Eagle Medium; αMEM: Alpha-Minimum Essential Medium; KO-DMEM: Knockout - Dulbecco’s Modified Eagle Medium; F12: Ham’s F12 Medium; LG-DMEM: Low-glucose Dulbecco’s Modified Eagle Medium; FBS: Fetal Bovine Serum; KSR: Knockout Serum Replacement.

Reproductive technologies such as *in vitro* fertilization (IVF), parthenogenetic activation (PA), and somatic cell nuclear transfer (SCNT) have facilitated the generation of diverse embryo sources for porcine genetic engineering and embryo research (Li et al., 2021; Redel et al., 2019). pESCs derived from embryos at developmental stages E5-E11 exhibit distinct pluripotency characteristics and culture requirements (Table 1; Supplementary Table S2; Figure 1). Crucially, the embryonic origin profoundly influences the developmental competence, pluripotency maintenance, and overall isolation efficiency of pESCs. Notably, the efficiency of establishing pESC lines from *in vivo*-derived embryos (∼10%) significantly exceeds that from *in vitro*-produced embryos (∼2.5%) (Vassiliev et al., 2010). This discrepancy is primarily attributed to morphological, epigenetic, and transcriptomic compromises induced by *in vitro* culture (IVC) systems.

**TABLE 1:** Source, Culture System, Morphological, and Pluripotency Parameters of porcine embryonic stem cells.

References	Cell sources	Feeder	Small molecules	Morphology	Passage	Karyotype	Pluripotency factors	Teratoma	Differentiation potency (*In vitro*)
Strojek et al. (1990)	*In vivo* matured E9-10	STO Porcine feeder	​	Refractile cells, no clone morphology	>4	-	-	-	-
Piedrahita et al. (1990a)	*In vivo* matured E7-8	Various	​	ES-like epithelial-like	>10	-	-	-	ES-like: Failed
Piedrahita et al. (1990b)	*In vivo* matured Days 7–8 post-estrus	STO PEF (no obtain)	-	ES-like epithelial-like cell line	32 >42	-	-	ES-like Failed	Epithelial-like: Vesicular structures
Wianny et al. (1997)	*In vivo* matured E7/E11	PFF	hLIF	Enlarged flattened	-	-	SSEA-1	-	-
Chen et al. (1999)	*In vivo* matured Day 6–8 post-estrus	STO	​	Round-shaped	>35	Normal	AP	-	Three germ layers
Li et al. (2003)	*In vivo* matured E7-9	MEF	rh-bFGF, rh-LIF	ES-like	9	-	AP	-	Various cell types
Li, et al. (2004b)	*In vivo* matured Day 7–9 post-estrus	MEF PEF STO	rh-bFGF, rh-LIF	Monolayer clone-like, cells resembling mouse morphology	9 (MEF) 5 (PEF) 1 (STO)	-	AP	-	Various cell types
Li, et al. (2004a)	IVF embryo 4-cell to blastocyst stages	MEF	rh-bFGF, rh-LIF	Monolayer clone-like	4	-	AP	-	Embryoid bodies formed and evolved into fluid-filled cystic structures after 8–10 days of culture
Yang et al. (2009)	*In vivo* matured Day 7	STO	​	Flatten clones	>90	Normal	Oct-4, AP, SSEA-4, TRA-1-60, TRA-1-81	Tiny	Three germ layers
Kim et al. (2010)	SCNT embryo	MEF	FGF	Flatten clones	>52 >48	Normal	AP, Oct-4, SSEA-1), SSEA-4, TRA-1–60, TRA-1–81	Failed	Three germ layers
Vassiliev et al. (2010)	*In vivo* matured E6-7 Day 7 *in vitro*-produced	MEF	bFGF, EGF, LIF, Activin A	Polygonal	>14	Normal	Oct4, Nanog, SSEA-1	-	Three germ layers
Tan et al. (2012)	Nuclear transfer embryo E5, E7, E9	MEF	rh-bFGF	Flatten clones	-	Normal	ES-like: AP^+^ D9: AP^-^	-	-
Vassiliev et al. (2011)	Cloned Embryos E7	MEF	bFGF, EGF, LIF, Activin A	Flatten clones	>15	Normal	Oct-4, Nanog	-	Three germ layers
Cheong et al. (2015)	IVF and PA embryo Day 7	MEF	bFGF	Dome-like morphology	-	-	​	-	Three germ layers
Siriboon et al. (2015)	PA and cloned embryos Day 7	STO MEF	FGF, LIF, Y-27632, 1,596–5	compact colonies	>28	Normal	AP, Oct4, Nanog, Sox2, Rex1	Failed	Three germ layers
Hou et al. (2016)	IVF embryos Day 6–7	STO MEF	bFGF, EGF, Activin-a	Flatten clones	STO >21 (10, 4 colonies) MEF≤10	Normal	AP, OCT4, NANOG, SOX2, SSEA-4, TRA-1-60, TRA-1-81	√	Three germ layers
Cha et al. (2018)	*In vivo* matured Day 6	MEF	EGF, LIF, BFGF	Well-defined, flattened	>20	Normal	SOX2, NANOG, TERT, OCT4, SOX2, TRA-1-60, TRA-1-81	-	Three germ layers
Zhang et al. (2019)	Cloned embryos Day 6	MEF	FGF, LIF, CHIR99021, PD0325901	Dome-like morphology	130	Normal	NANOG, OCT4, KLF-4, SOX-2, SSEA-1	-	Three germ layers
Yuan et al. (2019)	IVF embryos Day 7–8	MEF	IGF1, LIF, TGFβ1, FGF2, PD0325901, CHIR99021, SP600125, SB203580	Dome-like morphology	>45	-	POU5F1, NANOG, SOX2, AP	√	Three germ layers
Choi et al. (2019)	PA and IVF embryo Hatched blastocysts	MEF	hrFGF2, ActA, CH, IWR-1	Fatter	≥50	IVF-ES-12: 37, XY (extra chr. 17), Normal others	OCT4, SOX2, NANOG, SSEA1, SSEA4, TRA-1-6, TRA-1-81, AP	√	Three germ layers
Gao et al. (2019)	PA blastocysts	STO	CHIR99021, WH-4-023, XAV939, ACTIVIN A, LIF	Dome-like morphology	>20	Normal	OCT4, SOX2, and NANOG SSEA1, SSEA4	√	Three germ layers; PGC-like cells
Choi et al. (2020)	IVF and PA embryos	MEF	-	Large and flattened	>15	Normal	OCT4, SOX2, NANOG AP	√	-
Zhi et al. (2022)	*In vivo* matured embryos E5-E12	MEF	CHIR99021, IWR-1-endo, WH-4-023, LIF, Activin A, FGF-basic, Y27632	Dome-shaped	240	Normal	POU5F1, NANOG, SOX2 SSEA1, SSEA4, TRA-1-81, and TRA-1- 60, AP	√	Three germ layers
Lee et al. (2022)	PA embryos Day 7	MEF	bFGF	​	-	-	POU5F1, SOX2, NANOG	-	Three germ layers
Oh et al. (2022)	IVF embryos Hatched blastocysts	MEF	FGF, human LIF, AG1296, SB431542, PD0325901	Types A, B, C: single layers D: multiple layers	-	-	Type A: AP+, Types B, C: AP-, Type D: central part of the colony showed AP-positive	-	-
Fang et al. (2022)	SCNT embryos Day 7	MEF	​	Flat	-	-	NANOG, POU5F1, SOX2, c-Myc, Klf4, and TEAD4	-	-
Cai et al. (2023)	IVF cloned embryos Day 7	MEF	bFGF	Flat	-	-	AP	-	-
Choi H. et al. (2024)	IVF Day 6	MEF feeder-free	FGF2, IWR-1, WH-4-023, ROCKi	Dome-shaped	>50	Normal	POU5F1, NANOG, SOX2, SSEA-1, SSEA-4, TRA-1-60, except for TRA-1-81	√	Three germ layers
Xiang et al. (2024)	PA embryos	MEF	human IL-6, human sIL-6Rα, activin A, human IGF1, XAV939, IWR1, Y-27632	Dome-shaped	-	normal	POU5F, SOX2, NANOG, OTX2, E-Cadherin AP	√	Three germ layers
Ruan et al. (2024)	IVF and SCNT embryos Day 7	SNL76/7 feeders	CHIR-9902, WH-4-023, XAV939, Activin A, LIF	Dome-shaped	>48	Normal	OCT4, NANOG, SOX2, REX1, SSEA-4, SSEA-1	√	Derive porcine trophoblast stem cells (pTSCs)
Choi K. et al. (2024)	IVF and PA embryos Day 6	MEF Feeder-free	FGF2, ActA, CH, IWR-1, Y-27632, LDN-193189	Dome-shaped	>10	Normal	OCT4, SOX2, and NANOG SSEA1, and SSEA4	√	Three germ layers

BRL: buffalo rat liver cells; BRL-CM: conditioned medium of BRL; STO: murine embryonic fibroblasts; PEF: porcine embryonic fibroblasts; PUE: porcine uterine epithelial cells; MEF: murine embryonic fibroblasts; PH3A: An epithelial-like porcine embryo-derived cell line; IVF: in vitro fertilization; PA: parthenogenetic activation; SCNT: somatic cell nuclear transfer; DMEM: Dulbecco’s Modified Eagle Medium; FBS: fetal bovine serum; FCS: fetal calf serum; PS: porcine serum; CS: calf serum; HCS: human cord serum; hLIF: human leukemia inhibitory factor; rh-bFGF: recombinant human basic fibroblast growth factor; rh-LIF: recombinant human leukemia inhibitory factor; KSR: knockout serum replacement; aMEM: Alpha-Minimum Essential Medium; EGF: epidermal growth factor; Y-27632: ROCK, inhibitor; CHIR99021: GSK-3β inhibitor; PD0325901: ERK1/2 inhibitor; VC: Vitamin C; IGF1: Insulin-like Growth Factor 1; TGF β1: Transforming Growth Factor-β1; IWR-1: wnt inhibitor; WH-4-023: Lck/Src inhibitor; XAV939: Tankyrase inhibitor; AG1296: Tyrosine kinase inhibitor; SB431542: TGF-β, pathway inhibitor, A83-01: inhibitor of ALK5 (activin receptor-like kinase 5); LDN-193189: BMP (bone morphogenetic protein) signaling pathway inhibitor; IL-6: Interleukin-6; sIL-6Rα: Soluble Interleukin-6, receptor alpha subunit.

The reduced viability of *in vitro*-produced porcine embryos is evident early in development. Even a brief IVC period of 1–2 days following IVF can severely restrict their developmental capacity to reach the blastocyst stage and compromise full-term development post-transfer (Kikuchi et al., 1999). Compared to *in vivo* embryos, *in vitro*-produced blastocysts frequently exhibit higher incidences of apoptosis, lower total cell numbers, increased DNA damage, disorganized cytoskeletal structures, and irregular cell morphology (Hyun et al., 2003; Kidson et al., 2004; Long et al., 1998). Furthermore, comparative analysis of chromatin and nucleolar dynamics reveals that *in vitro*-produced embryos suffer from aberrant chromatin interactions at the two-cell stage, delayed chromatin decondensation and nucleolar formation at the four-cell stage, and the absence of a heterochromatin halo, ultimately leading to defective lineage segregation at the blastocyst stage (Deshmukh et al., 2012).

Beyond cellular morphology, the epigenetic landscape of *in vitro* embryos differs markedly from their *in vivo* counterparts, presenting a major barrier to the establishment and long-term stability of pESC lines (Zhang, et al., 2014a). *In vivo* embryos undergo a natural progression of global demethylation from the 2-cell to the 8-cell stage, followed by lineage-specific *de novo* methylation at the blastocyst stage, characterized by a hypermethylated inner cell mass (ICM) and a hypomethylated trophectoderm (TE) (Deshmukh et al., 2011). In contrast, IVF embryos display abnormally high methylation levels at the one-cell stage and fail to establish lineage-specific methylation patterns at the blastocyst stage (Deshmukh et al., 2011). SCNT embryos face even more severe epigenetic hurdles during zygotic genome activation (ZGA), exhibiting abnormal H3K4me3, H3K9me3, and H3K27me3 modifications alongside stochastic reprogramming inefficiencies (Liu et al., 2022b). Consequently, SCNT embryos often partially retain somatic methylation signatures, resulting in a hybrid epigenetic state that blends *in vivo*-like and PA-like patterns (Liu et al., 2022b; Deshmukh et al., 2011). Understanding and correcting these origin-specific epigenetic defects is imperative to overcome the high inter-laboratory variability and differentiation bias frequently observed during prolonged pESC culture.

**FIGURE 2:**
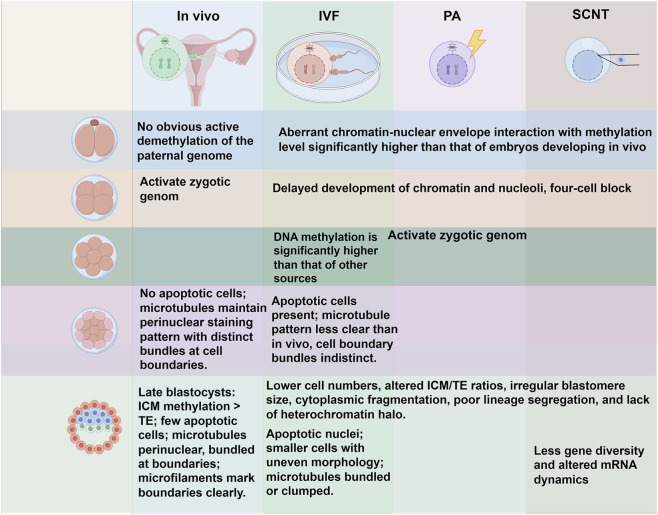
Comparative analysis of chromatin and nucleolar dynamics, epigenetic reprogramming, and developmental characteristics between *in vivo* developed and *in vitro* produced porcine embryos. Significant differences were observed in chromatin and nucleolar dynamics of porcine embryos developed *in vivo* and those produced through *in vitro* fertilization (IVF), parthenogenetic activation (PA), or somatic cell nuclear transfer (SCNT). *In vitro* produced embryos exhibited abnormal chromatin dynamics, including aberrant interactions at the two-cell stage, delayed decondensation and nucleolar development at the four-cell stage, and defective lineage segregation at the blastocyst stage. They also lacked a heterochromatin halo, suggesting impaired development. Specifically, SCNT embryos exhibited aberrant epigenetic reprogramming during zygotic genome activation, with abnormal modifications of H3K4me3, H3K9me3, and H3K27me3 compared to fertilized embryos. Additionally, *in vitro* embryonic development was associated with increased DNA damage, disorganized cytoskeletal structures, and more irregular cell morphology when compared with *in vivo* embryos.

Transcriptional profiling further highlights the distinct molecular nature of embryos and stem cell lines derived from different origins (Figure 2). Single-cell RNA sequencing and lineage tracing have elucidated the molecular basis of pluripotency across diverse embryonic sources (Zhi et al., 2022; Kinoshita et al., 2021). For instance, *in vivo* embryos successfully initiate ZGA at the 4-cell stage and express unique gene profiles associated with fatty acid metabolism (Cao et al., 2014). Conversely, *in vitro* embryos, particularly SCNT-derived ones, often show delayed ZGA at the 8-cell stage (Cao et al., 2014). Additionally, PA-derived embryos exhibit reduced gene diversity and altered mRNA dynamics, which negatively impact essential processes such as mRNA splicing and energy metabolism (Fan et al., 2024). These transcriptomic variations reflect unique pluripotency stages and adaptive changes induced by specific culture conditions.

To mitigate the developmental barriers of *in vitro* embryos, epigenetic modifiers have been successfully employed. The supplementation of culture media with compounds such as valproic acid (VPA) and oxamflatin has been reported to significantly enhance the developmental potential of porcine SCNT embryos both *in vitro* and *in vivo* (Huang et al., 2011; Hou et al., 2014). Given that the derivation efficiency and quality of pESCs are intrinsically tied to their embryonic source, it is evident that a “one-size-fits-all” culture system is inadequate. Generally, *in vivo*-matured embryos yield pESCs with superior pluripotency and differentiation potential. IVF embryos preserve genetic integrity but require highly optimized culture conditions to achieve comparable quality, while PA embryos, lacking a paternal genome, inherently display reduced pluripotency marker expression and differentiation capacity. Therefore, tailoring derivation protocols and culture systems to accommodate the specific epigenetic and metabolic requirements of each embryonic origin remains a critical frontier for advancing pESC applications in developmental biology and agriculture.

### 3.2 Feeder cells in porcine embryonic stem cell isolation

Feeder cells are crucial for maintaining pESC pluripotency by providing an essential niche composed of extracellular matrix (ECM) proteins and growth factors (e.g., bFGF, TGFβ, Activin A) (Ludwig et al., 2006; Steiner et al., 2010). Early experiments assessing various feeder layers demonstrated that porcine embryo-derived cell lines required specific support, such as primary mouse embryonic fibroblasts (MEFs) or STO cells, to promote attachment and proliferation (Piedrahita et al., 1990a).

While MEFs remain the standard, their limited proliferative capacity and batch-to-batch variability can affect experimental reproducibility (Karatza and Shall, 1984). The STO (Sandoz inbred Swiss Mouse (SIM) thioguanine-resistant ouabain-resistant) cell line, an immortalized line derived from mouse SIM embryonic fibroblasts, has been used as an alternative feeder cell line for ESC culture in various species (Park et al., 2004; Park et al., 2015) Although STO cells express lower levels of ECM proteins compared to MEFs (Ahn et al., 2023), offering a more scalable option for agricultural applications.

Recent efforts have also focused on developing feeder-free culture systems using small molecule inhibitors (e.g., the BMP inhibitor LDN-193189) to replace feeder signals (Choi K. et al., 2024). However, porcine pluripotent cells exhibit distinct niche requirements compared to human ESCs; consequently, feeder-free conditions often fail to support robust, long-term self-renewal without strict control of ECM coatings (Choi K. et al., 2024; Choi et al., 2019), highlighting an ongoing technical barrier in the field.

### 3.3 Medium optimization in the derivation of porcine embryonic stem cells

The optimization of culture media is a cornerstone in the derivation and sustainable maintenance of pESCs. This is particularly critical in agricultural biotechnology, where genetically stable and pluripotent cell lines are prerequisites for advancing livestock breeding, germline modification, and the production of disease-resistant pigs. The selection of an appropriate basal medium represents the first critical step. A recent comprehensive evaluation revealed that DMEM/F10 and DMEM/F12 significantly outperform α-MEM in supporting ESC-like colony formation (Choi H. et al., 2024), largely due to the presence of essential inorganic components required for stem cell maintenance (Fathi and Farahzadi, 2018; Díaz-Tocados et al., 2017; He et al., 2021; Celauro et al., 2017).

Building upon basal formulations, a diverse array of small molecules and growth factors has been employed to precisely regulate key signaling pathways (Table 2). Rather than relying on a single pathway, recent protocols utilize complex cocktails targeting multiple networks simultaneously. These include TGF-β modulators (e.g., Activin A, SB431542) to suppress premature differentiation, alongside inhibitors of ROCK, MAPK/ERK, PI3K/AKT, and epigenetic modifiers (e.g., HDAC inhibitors) to stabilize the pluripotent state (Choi et al., 2019; Zhi et al., 2022; Yuan et al., 2019; Vassiliev et al., 2011; Zhang et al., 2019; Gao et al., 2019). Notably, the signaling requirements for porcine pluripotency diverge significantly from those of rodents. For instance, the traditional “2i” condition (dual inhibition of MEK and GSK3β), which robustly captures the naïve ground state in murine ESCs, frequently induces severe differentiation or apoptosis in porcine cells (Petkov et al., 2014; Li et al., 2020).

**TABLE 2:** Summarizes small molecules used for the isolation of porcine embryonic stem cells.

Pathway	Small molecules or cytokines	Function	Change during the porcine embryonic development process
JAK/STAT3	Leukemia Inhibitory Factor (LIF) IL-6 and sIL-6 Rα	LIF stimulates Stat3 *via* the LIF receptor-interleukin 6 signal transducer (gp130) complex, and Jak kinase. Activated Stat3 dimerizes, translocates into the nucleus, and regulates expression of downstream target genes as a transcription factor. The LIF/Stat3 pathway is essential for the self-renew ability of mouse ES cells (Niwa et al., 1998; Matsuda et al., 1999) IL-6 binds to sIL-6R, and the IL-6/sIL-6R complexes interact with membrane-bound gp130 (Tian et al., 2011) to induce activation of the JAK/STAT signaling pathway (Rivera-Soto et al., 2020)	Hub genes in the JAK/STAT3 pathway are highly expressed from E4 early morulae to E6 inner cell masses, declining after E7 epiblast formation (Zhi et al., 2022)
Activin/Nodal	Activin A	Activin A is a key ligand in the Activin/Nodal pathway, maintaining pluripotency in hESCs and hiPSCs, especially in feeder-free conditions (Beattie et al., 2005; Tomizawa et al., 2013; Theunissen et al., 2014)	Activin A receptors are highly expressed in epiblasts from E7 to E10 (Zhi et al., 2022)
TGF-β	SB431542 A83-01 LDN-193189 TGFβ1	SB - 431,542 is used to prevent phosphorylation of Smad2/3 and the growth inhibition induced by TGF-β (Tojo et al., 2005). A83-01 is an inhibitor of ALK4, 5, 7 receptors, and ALK5 is the key mediator of TGFβ signalling (Tojo et al., 2005). TGFb1, Activin A, and Nodal ligands might play a role in the activation of the TGFb1/Activin/Nodal pathway in hESCs under mechanical strain (Saha et al., 2008)	Smad1, Smurf1, and Id4 in the TGF-pathway were highly expressed in pig ICM (Cao et al., 2014)
FGF/ERK	FGF	FGF signaling is critical for stem cell pluripotency, mediated by RAS-MAPK, PI3K-AKT, PLCγ, and STAT pathways (Mossahebi-Mohammadi et al., 2020; Stavridis et al., 2010; Fathi et al., 2017; Tang et al., 2019). It regulates proliferation, differentiation, and embryonic development (De. FGF maintains pluripotency by regulating key transcription factors like Oct4, Sox2, and Nanog (Rizzino.	FGF2 receptors are highly expressed in epiblasts from E7 to E10 (Zhi et al., 2022)
Wnt/β-catenin	CHIR99021 IWR-1-endo	CHIR99021 stabilizes β-catenin by inhibiting GSK-3β, activating Wnt/β-catenin pathway, influencing proliferation, differentiation, and self-renewal (Sato et al., 2004; Kielman et al., 2002; Reya et al., 2003; Zhao et al., 2007; Atlasi et al., 2014; Nusse, 2008) IWR-1-endo inhibits Wnt/β-catenin signaling	Significantly increased activity during the epiblast-to-ectoderm transition from E10 to E11 (Zhi et al., 2022)
ROCK	Y-27632	Y-27632 inhibits ROCK1 and ROCK2, preserving the integrity of hESCs. It influences proliferation of pluripotent stem cells, aiding recovery of frozen-thawed hESCs and hiPSCs (Li et al., 2009; Martin-Ibañez et al., 2008; Watanabe et al., 2007; Yu et al., 2007; Takahashi et al., 2007; Okita et al., 2007; Claassen et al., 2009)	​
ERK1/2	PD0325901	The role of ERK in maintaining stemness is controversial. In different cellular systems, it may not contribute to cell proliferation (Ajenjo et al., 2000), may induce differentiation (Racke et al., 1997), or may maintain stemness (Ciccarelli et al., 2016). In glioblastoma (GB), ERK expression is related to stem cells (Riddick et al., 2017), but the interconnection between PI3K and ERK signaling complicates the distinction of their effects on downstream proteins	​
Lck/Src	WH-4-023	WH-4-023, a dual Lck/Src inhibitor, improves viability and morphology in pluripotent stem cells, affecting OCT4-ΔPE-GFP reporter activity (Theunissen et al., 2014)	​
PDGF	AG1296	AG1296, a PDGFRβ inhibitor, influences ES cell differentiation and survival, blocking cyclic strain-induced effects, and improving iPSC-EC survival (Shimizu et al., 2008; Gu et al., 2021)	​

EGF: epidermal growth factor; Y-27632: ROCK, inhibitor; CHIR99021: GSK-3β inhibitor; PD0325901: ERK1/2 inhibitor; IGF1: Insulin-like Growth Factor 1; TGF β1: Transforming Growth Factor-β1; IWR-1: wnt inhibitor; WH-4-023: Lck/Src inhibitor; XAV939: Tankyrase inhibitor; AG1296: Tyrosine kinase inhibitor; SB431542: TGF-β, pathway inhibitor, A83-01: inhibitor of ALK5 (activin receptor-like kinase 5); LDN-193189: BMP (bone morphogenetic protein) signaling pathway inhibitor; IL-6: Interleukin-6; sIL-6Rα: Soluble Interleukin - 6, Receptor alpha subunit.

Recognizing this species-specific divergence, researchers have focused on the FGF/ERK pathway, which is pivotal for epiblast maintenance in both pigs and humans (Choi et al., 2018; Xu et al., 2001; Zhao and Jin, 2017). Recent derivation strategies have utilized combinations of FGF2, IWR-1 (a canonical Wnt inhibitor) and CHIR99021 (a GSK3β inhibitor) to promote pESCs derivation (Choi H. et al., 2024). In this context, FGF2 drives epiblast proliferation between embryonic days E7 and E10 (Zhi et al., 2022), while the delicate balance between IWR-1 and CHIR stabilizes the pluripotent states (Martins-Neves et al., 2018; Kim et al., 2013). However, maintaining this equilibrium is highly sensitive; lower concentrations of CHIR (e.g., 0.5 µM) have been associated with early differentiation (Choi H. et al., 2024). Mechanistically, this sensitivity mirrors the developmental dynamics of porcine embryos, where the epiblast-to-ectoderm transition becomes governed by Wnt/β-catenin signaling starting at E10. A landmark protocol, termed 3i/LAF, was developed based on a large-scale single-cell transcriptomic atlas of pig embryos from E0 to E14. This formulation chemically inhibits WNT-related signaling (via IWR-1 and CHIR99021) while providing growth factors that activate the FGF/ERK, JAK/STAT3, and Activin/Nodal pathways. Using this defined cocktail, stable pregastrulation epiblast stem cell lines (pgEpiSCs) were successfully derived from E10 epiblasts, maintaining a normal karyotype and pluripotency for over 240 passages (Zhi et al., 2022). In parallel, a chemically defined medium termed 4FIXY (containing activin A, IGF1, IL-6, XAV939, IWR1, and Y27632) was established to derive pESCs from parthenogenetic blastocysts. Notably, these pESCs maintained in 4FIXY exhibit a primed pluripotent state and, upon transfer to a 3D two-step differentiation system, can efficiently self-organize into porcine blastoids that recapitulate the morphology, cell lineage composition (EPI, TE, HYPO), and single-cell transcriptome of natural blastocysts (Xiang et al., 2024). Consequently, Wnt inhibitors like XAV939 and IWR-1 are indispensable not only for sustaining pluripotency but also for preventing the spontaneous conversion of pESCs into extraembryonic endoderm (XEN) cells (Zhi et al., 2022; Wang et al., 2025).

To counteract residual differentiation tendencies, the incorporation of SRC inhibitor WH-4-023 facilitated the establishment of the highly optimized pESC-FIW line. This line exhibits superior clonal efficiency, and the capacity for stable long-term culture via single-cell passaging (Lee et al., 2019; Collier and Rugg-Gunn, 2018; Choi H. et al., 2024). However, its potential to generate high-contribution chimeras or support germline transmission remains untested. Despite these advancements, the FIW medium remains insufficient to support pluripotency under strictly feeder-free conditions, even when supplemented with Act A or TGFβ1 (Choi H. et al., 2024). This contrasts sharply with human ESCs and iPSCs, which readily adapt to feeder-free environments (Beattie et al., 2005; Tomizawa et al., 2013; Vallier et al., 2009). The recalcitrance of pESCs to feeder-free culture may stem from species-specific extracellular matrix requirements; specific integrin heterodimers (α5β1, α9β1, and αVβ1) have been identified on porcine primed ESCs, which mediate essential adhesion to fibronectin, tenascin C, and vitronectin (Kim et al., 2018).

Understanding these unique biochemical requirements is aided by classifying porcine pluripotency into naïve, formative, and primed states. While optimized media have improved derivation efficiencies, several critical bottlenecks remain. To synthesize the current state of the field and highlight the most pressing barriers hindering both biomedical and agricultural translation, we have summarized the major unresolved challenges in porcine stem cell biology in Table 3 (Shi et al., 2025; Choi et al., 2019).

**TABLE 3:** Major unresolved challenges in porcine embryonic stem cell biology and translational applications.

Challenge category	Specific biological/Technical barrier	Current status and impact	Future directions/Potential solutions
Naïve Pluripotency	Species-specific resistance to conventional naïve culture conditions (e.g., “2i” media)	Most pESC/piPSC lines remain locked in a primed or formative state. True naïve pluripotency is transient and difficult to stabilize	Deconvolution of porcine-specific pluripotency networks; high-throughput screening of novel small-molecule inhibitor cocktails
Germline Competence	Inability to consistently generate high-contribution, germline-transmitting chimeras	Hinders the creation of genetically modified pig models for agriculture and biomedicine. Serves as the ultimate missing proof of authentic pluripotency	Optimization of host embryo complementation techniques; correction of epigenetic roadblocks prior to chimera generation
Epigenetic Instability	Progressive erosion of X-chromosome inactivation, abnormal imprinting patterns, and retention of somatic memory (in SCNT lines)	Leads to unpredictable differentiation bias, reduced developmental competence, and tumorigenic risks during long-term culture	Leads to unpredictable differentiation bias, reduced developmental competence, and tumorigenic risks during long-term culture
Reproducibility	High inter-laboratory variability; heavy reliance on undefined feeder cells (MEFs/STO) and serum components	Complicates the comparison of results across studies and limits the standardization of pESC/piPSC derivation protocols	Development of fully defined, chemically formulated, feeder-free culture matrices tailored to porcine-specific integrin profiles
Scalability for Agriculture	High cost of defined media; loss of stemness/differentiation potential during large-scale expansion in bioreactors	Poses a major economic and technical barrier for industrial-scale applications, particularly in cultured meat production	Engineering cost-effective, serum-free media formulations; development of edible scaffolds that support scalable 3D expansion while maintaining stemness

Moving forward, agriculturally focused research should integrate advanced multi-omics tools, such as single-cell RNA sequencing to characterize culture heterogeneity, alongside CRISPR/Cas9 for targeted genetic improvements. Concurrently, standardizing ethical and regulatory frameworks will be essential as pESC technologies transition from *in vitro* laboratory models to practical, transformative applications in modern livestock biotechnology.

## 4 Porcine induced pluripotent stem cells

Porcine induced pluripotent stem cells (piPSCs) represent a promising alternative to pESCs for research and agricultural applications. Generated by reprogramming somatic cells through the forced expression of key transcription factors, typically OCT4, SOX2, KLF4, and c-MYC, piPSCs offer a renewable source of pluripotent cells while circumventing some of the ethical and technical limitations associated with embryo-derived pESCs. The development of piPSCs began in 2009, with early lines showing characteristics similar to human ESCs, including a normal karyotype, expression of pluripotent markers, and the capacity to differentiate into all three germ layers (Ezashi et al., 2009; Wu et al., 2009; Esteban et al., 2009). However, these early piPSCs generally required continuous exogenous transgene expression to maintain their pluripotency, representing a major limitation for downstream applications.

### 4.1 Advances in reprogramming strategies and culture systems

To improve reprogramming efficiency and obtain transgene-silenced or transgene-free piPSCs, various strategies have been explored. These include the use of different starting cell types, novel combinations of reprogramming factors (encompassing transcriptional and epigenetic modulators), improved delivery systems, and optimized culture media formulations (Neira et al., 2024). Cell types successfully employed for reprogramming include embryonic fibroblasts (Conrad et al., 2023), mesenchymal stem cells (Liu et al., 2012), adipose-derived stem cells (Zhang et al., 2014b), and Sertoli cells (Setthawong et al., 2019).

Regarding delivery systems, while, early protocols often employed integrating lentiviral vectors to accelerate colony formation (Yu et al., 2022), recent efforts have shifted toward non-integrating methods, including adenovirus, Sendai virus (Baek et al., 2023), small molecules (Chen et al., 2021), and episomal vectors (Li et al., 2018; Yoshimatsu et al., 2021; Zhu, et al., 2023b; Conrad et al., 2023), to avoid insertional mutagenesis. Notably, a recent step-by-step protocol utilizing episomal plasmids encoding a comprehensive factor cocktail (POU5F1, SOX2, NANOG, KLF4, SV40LT, c-MYC, LIN28A, and microRNA-302/367) enables the efficient derivation of transgene-free piPSCs in approximately 4 weeks, directly addressing the historical transgene retention problem (Conrad et al., 2024).

In parallel, extensive optimization of culture media has been critical for piPSC derivation and maintenance. Formulations typically utilize N2B27 or KOSR basal media supplemented with specific signaling pathway inhibitors (e.g., targeting GSK3, MEK, and p38 MAPK) and growth factors (e.g., LIF, FGF2, and Activin) (Yuan et al., 2019; Zhou et al., 2023). A landmark chemically defined strategy consisted of a two-stage approach: an induction medium (iCD3) that first promotes epithelial-like precursors, followed by a maintenance cocktail (LACID) containing IWR-1. Integration-free piPSCs derived using this iCD3/LACID system with episomal vectors exhibited improved karyotype stability, core pluripotency gene expression upon episome loss, and sustained expansion for over 30 passages without doxycycline or feeder cells (Shi et al., 2026). Note: A comprehensive review systematically catalogues piPSC derivation methodologies reported between 2009 and early 2024; further methodological details are not reiterated here (Neira et al., 2024).

Beyond factor delivery and culture conditions, the molecular identity of the reprogramming factors critically influences outcomes. A comparative study demonstrated that the species origin (porcine, bovine, or murine) of exogenous transcription factors profoundly impacts both the efficiency of piPSC generation and the resultant cellular properties (Zhou et al., 2023). Further mechanistic insight revealed that overexpression of the chromatin remodeler BRG1 enhances piPSC generation by facilitating glycolytic reprogramming through the PI3K/AKT signaling pathway. BRG1 upregulates glycolysis-related genes (HK2, PKM2, PFK-1), increases glycolytic metabolites, and decreases H3K9me3 enrichment at pluripotency and glycolysis-related gene promoters, thereby establishing a direct link between epigenetic remodeling and metabolic reprogramming in the porcine system (Ren et al., 2024).

### 4.2 Key unresolved barriers

Incomplete silencing of exogenous transcription factors and insufficient activation of endogenous pluripotency networks remain major limitations for some piPSC lines. Additionally, the long-term stability and reproducibility of piPSCs present significant challenges. Variability between laboratories is frequently observed, often manifesting as epigenetic instability, gradual loss of pluripotency markers during extended culture, or lineage biases during differentiation. Historically, the establishment of stable porcine pluripotent stem cell lines capable of passing stringent pluripotency tests has proven challenging, primarily because culture conditions optimized for murine or primate PSCs are not directly transferable to the porcine system (Neira et al., 2024).

To address these fundamental scientific challenges, it is crucial to systematically understand the porcine-specific pluripotency network, which differs significantly from well-characterized rodent and human models (Brevini et al., 2012). Furthermore, the conceptual framework regarding pluripotent states, specifically the distinction between naïve and primed pluripotency, remains less clearly defined in pigs than in mice and humans. A recent comprehensive review systematically surveyed the features of totipotent, naïve, and formative pluripotent stem cells across species. Highlighting nine porcine stem cell studies, the review noted that current *in vitro* cell models exhibit distinct epigenetic and transcriptional differences from *in vivo* embryos, thus imperfectly recapitulating embryonic development (Hua et al., 2025).

Whereas murine pluripotency relies heavily on the LIF/STAT3 pathway and human pluripotency is governed by FGF2/NODAL signaling, the porcine pluripotency network exhibits distinct regulatory mechanisms (Hall and Hyttel, 2014). Accumulating evidence underscores the predominant role of the FGF/ERK and WNT/β-catenin pathways in establishing and sustaining porcine pluripotency, while the LIF/STAT3 pathway appears to play a relatively minor or even dispensable role (Brevini et al., 2012). For example, the functionally advanced piPSCs cultured in the LACID system are acknowledged by the authors to reside in a primed-like pluripotent state (Shi et al., 2026). Collectively, these observations indicate that porcine pluripotent cells are locked in a primed-like state due to a combination of: (i) a predominant reliance on FGF/ERK signaling rather than LIF/STAT3; (ii) incomplete X-chromosome reactivation; and (iii) aberrant DNA methylation patterns that resist reprogramming toward naïve pluripotency. Achieving a stable naïve state in ungulates is currently hindered by unresolved, species-specific epigenetic barriers, such as incomplete X-chromosome reactivation and aberrant DNA methylation patterns (Hua et al., 2025). Overcoming these barriers remains a key unresolved question in the field.

### 4.3 Expanding functional utility

Despite foundational challenges, iterative improvements in cell line quality have markedly expanded the experimental and translational utility of piPSCs. Recent advances have enabled the generation of genetically engineered lines for specific applications. For instance, CRISPR/Cas9-mediated generation of SOX2-ZsGreen reporter lines now facilitates the real-time monitoring of pluripotency status (Reddy Yeddula et al., 2024). Similarly, disease modeling has been advanced by the derivation of CD163-knockout piPSCs, which lack the key receptor for PRRSV infection. These lines retain multilineage differentiation potential while providing a reliable *in vitro* platform for investigating host-pathogen interactions (Zhao et al., 2026).

Furthermore, piPSCs are increasingly being leveraged for regenerative medicine and tissue engineering. Multiple recent studies have demonstrated the efficient differentiation of piPSCs into functional vascular endothelial cells (piPSC-ECs). This process can be significantly enhanced by initial hypoxic microenvironments (Li et al., 2024) or the use of specific substrates like laminin-411 in 3D scaffolds (Liao et al., 2025). Crucially, these piPSC-ECs have been successfully utilized to produce tissue-engineered vascular conduits (TEVCs) that maintain endothelial functionality and prevent thrombosis when implanted *in vivo*, highlighting the potential of piPSCs for preclinical cardiovascular therapies (Batty et al., 2025).

In ophthalmology, piPSCs have been successfully directed to form complex, photoreceptor-dominant retinal organoids containing major neural retina cell types with highly organized neuroepithelium (Edwards et al., 2025). The scalable production of such organoids provides a vital pathway for developing conspecific (pig-to-pig) transplantation models for retinal degenerative diseases, thereby avoiding the immunological incompatibilities inherent to human xenografts, a strategic priority for translating advanced retinal therapies (Bellingrath et al., 2024).

Beyond specific lineage differentiation, the overall functional competency of piPSCs has reached new milestones. Advanced lines, such as those maintained in the LACID system, have overcome longstanding developmental barriers by demonstrating the capacity to form blastoids, contribute to chimeric blastocysts, and serve as viable nuclear transfer donors for blastocyst production (Shi et al., 2026).

Together, piPSC technology has evolved from transgene-dependent lines with limited utility to sophisticated, transgene-free systems with demonstrated functional competence in disease modeling, tissue engineering, and embryo-like structure generation. Nevertheless, a deeper understanding of the porcine pluripotency regulatory network remains essential for overcoming species-specific barriers and realizing the full translational potential of these cells.

## 5 Porcine germline stem cells

### 5.1 Spermatogonial stem cells

Spermatogonial stem cells (SSCs) are the foundational adult stem cells of male mammalian reproduction, uniquely capable of both self-renewal and differentiation to produce spermatozoa. Located on the basement membrane of seminiferous tubules, porcine SSCs (pSSCs) serve as the sole conduit for transmitting genetic information to subsequent generations (de Rooij, 1973; Huckins, 1971; Lok et al., 1982).

#### 5.1.1 Molecular identity and markers

A fundamental obstacle in pSSC research has been the lack of definitive, species-specific molecular markers, which directly limits the purity of isolated populations and the reproducibility of functional assays across laboratories. Unlike in mice, where a well-characterized hierarchy of markers (e.g., ID4, GFRα1, PLZF, c-KIT) distinguishes stem from progenitor spermatogonia, the porcine marker landscape remains fragmentary and less rigorously validated.

Various surface markers, including CD14, CD49f, CD99, PODXL2, and SSEA4, have been investigated to isolate early pSSCs and separate them from differentiating populations (Park et al., 2019; Maki et al., 2009; Zhang et al., 2020; Zhang et al., 2021). However, the field currently lacks consensus on a core marker panel, as none of these markers is exclusively expressed in pSSCs, and their expression can vary with developmental stage, culture history, and donor age, underscoring the need for systematic cross-laboratory validation.

Recent single-cell transcriptomic analyses in Shaziling pigs have begun to address this gap by identifying novel candidate markers, including MDGA2, TENM2, MAGI2, PRKG1, GPC6, LRP1B, and CTNNA2 (Tang et al., 2024). In parallel, ZBTB16 (PLZF) has been shown to regulate the adhesion and proliferation of porcine immature spermatogonia (Cui et al., 2025), and studies employing single-cell resolution have provided transcriptional atlases of porcine spermatogenesis (Zhang et al., 2021; Wang et al., 2024). These transcriptomic resources, if integrated with functional transplantation assays, the gold standard for SSC identity, hold considerable promise for defining a validated porcine SSC signature.

#### 5.1.2 *In vitro* culture systems

Establishing robust *in vitro* culture systems for pSSCs is a prerequisite for downstream applications, yet published protocols vary substantially, making direct comparisons difficult. Rather than a standardized approach, diverse strategies have been employed: while some systems rely on neonatal Sertoli cell feeder layers combined with GDNF, GFRα1, bFGF, and IGF1 to maintain pSSCs (Zhang et al., 2017), others have successfully utilized feeder-free conditions, low-serum media (Zheng et al., 2013).

Notably, the selective GSK-3β inhibitor SB216763 supported the long-term culture (55 days) of porcine spermatogonial cells, enhancing proliferation, survival, and the expression of undifferentiated markers (Zhou et al., 2026). However, the lack of clonal derivation and transplantation assays highlights a broader issue: “successful culture” is defined differently across studies (by morphology, marker expression, or transplantation efficiency), with few reports providing quantitative functional validation. Moreover, prolonged culture invariably leads to gradual loss of stemness, a phenomenon well documented in other species but only beginning to be systematically addressed in pigs. Development of chemically defined, xeno-free culture platforms, informed by an improved understanding of the porcine SSC niche, including the specific contributions of Sertoli cells (You et al., 2026), is therefore a priority.

#### 5.1.3 Epigenetic and transcriptional regulation

Understanding the molecular mechanisms governing pSSC self-renewal and differentiation is essential for the rational design of culture systems and genetic manipulation strategies. This area remains considerably understudied in pigs compared with rodents and primates. The histone methyltransferase SETDB1, which catalyzes H3K9 trimethylation, has been shown to regulate the proliferative activity and adhesion of porcine spermatogonial stem/progenitor cells, at least in part through transcriptional repression of matrix metalloproteinases *MMP3* and *MMP10* (Liu et al., 2022a). The SDF-1/CXCR4 chemokine axis maintains porcine prospermatogonia in an undifferentiated state by activating the PI3K-AKT-AP-1 signaling cascade, which upregulates the transcriptional repressor PLZF while also modulating the expression of the differentiation mediator DMRT1 (Wang et al., 2025a). At the three-dimensional chromatin level, Hi-C analyses have revealed that differentiating spermatogonia exhibit a more relaxed chromatin architecture with weakened compartmentalization and fewer topologically associating domains compared with undifferentiated spermatogonia, indicating that large-scale chromatin decondensation is preprogrammed before meiotic entry (Zheng et al., 2022b). Furthermore, transcriptome-wide m^6^A profiling has uncovered extensive epitranscriptomic remodeling during porcine spermatogenesis, with m^6^A marks enriched on genes encoding metabolic enzymes and key fate determinants such as SETDB1, FOXO1, and FOXO3 (Liu et al., 2023b). Together, these findings establish an initial framework, but systematic functional analyses, including conditional knockout models and lineage-tracing studies, are still needed to fully define the core regulatory circuitry of pSSCs.

#### 5.1.4 Applications in genetic breeding and translational challenges

SSCs also offer potential applications in genetic breeding. Conceptually, SSCs from genetically superior males can be transplanted into recipient males to disseminate elite genetics, providing an alternative or complement to traditional artificial insemination. This technique has been successfully demonstrated in several livestock species. Immortalized pSSC lines may also serve as tools for genetic manipulation and cell-based therapies (Zheng et al., 2020).

In considering immortalization strategies for pSSCs, which would be necessary for scalable applications, the experience from other species provides instructive guidance. While CRISPR/Cas9-mediated immortalization (e.g., through knockout of TP53 or overexpression of hTERT) can achieve rapid results, such genetic engineering approaches carry potential risks of off-target effects and raise regulatory and consumer acceptance concerns, particularly for food applications. In contrast, spontaneous immortalization, which occurs through natural chromosomal rearrangements and epigenetic changes during extended passaging, has been shown in chicken fibroblasts to yield cell lines with remarkable long-term genetic stability and no tumorigenic potential, even after more than 600 population doublings and over 1.5 years of continuous culture (Pasitka et al., 2023). Comprehensive safety assessments including karyotype analysis, copy number variation profiling, TP53 SNV analysis, comet assays for DNA repair capacity, soft agar colony formation assays, and analysis of cancer-associated mutations all supported the safety profile of spontaneously immortalized lines (Guo et al., 2026; Pasitka et al., 2025). Whether similar spontaneously immortalized pSSC lines can be established remains an open question, but this paradigm offers a potentially safer and more regulator-friendly path for generating porcine germline cell resources, should the field wish to avoid the complexities associated with genetic modification in food-animal applications.

However, the translational gap between experimental proof-of-concept and practical application is considerable. First, the scalability of SSC derivation and expansion to levels sufficient for agricultural breeding programs has not been demonstrated, as current protocols generate limited cell numbers and are labor-intensive. Second, the reproducibility of transplantation outcomes is affected by multiple variables, including recipient preparation, cell dose, and delivery method, and the efficiency of donor-derived spermatogenesis in pigs remains poorly characterized. Third, robust regulatory frameworks for food products derived from stem cell-based breeding technologies have yet to be developed. Moreover, environmental toxicants relevant to agricultural settings, such as hexavalent chromium, which has been shown to induce mitophagy and ferroptosis in pSSCs (Li et al., 2026), may impact SSC quality and function in ways that are not yet fully appreciated. Addressing these challenges will require integrated efforts spanning cell biology, bioprocess engineering, and regulatory science before the practical value of pSSCs in livestock improvement can be fully realized.

### 5.2 The debate on female germline stem cells in pigs

Mammalian ovaries have traditionally been regarded as possessing a finite oocyte pool established before or shortly after birth, with no capacity for postnatal oocyte renewal. Consequently, the notion of female germline stem cells (FGSCs) capable of sustaining postnatal oogenesis remains a topic of intense debate (Huang and Ye, 2025). Proponents point to the isolation of VASA-positive cells with germline characteristics from multiple mammalian species. Conversely, critics emphasize the failure of independent replication (Byskov et al., 2011), the lack of definitive *in vivo* lineage-tracing evidence (Zhang et al., 2014), and the possibility that putative FGSCs may represent *in vitro* artifacts or misidentified somatic cells (Huang and Ye, 2025). A substantial body of evidence maintains that neo-oogenesis does not occur naturally in adult ovaries and that the oocyte reserve is strictly fixed at birth (Dunlop et al., 2014), underscoring the limitations and uncertainties of the FGSC concept.

Despite these controversies, several studies have reported the isolation of FGSC-like cells from porcine ovaries. Putative FGSCs were first characterized in juvenile porcine ovaries as VASA-positive cortical cells expressing pluripotency and germline markers (Bai et al., 2013). Subsequent studies successfully isolated these cells from sexually mature sows, demonstrating their ability to differentiate into oocyte-like cells (OLCs) under defined conditions (Hou et al., 2018), and from both pre-pubertal and adult ovaries using VASA antibody-based magnetic sorting (Nguyen et al., 2019). Most recently, pFGSCs isolated from 1-day postpartum piglet ovaries were shown to express key pluripotency and germline markers (Oct4, C-kit, Vasa, Stella, Ifitm3, and Dazl), with a small proportion (2.81% ± 0.76%) spontaneously differentiating into OLCs. While co-supplementation with 5% porcine follicular fluid (PFF) and 1 μM retinoic acid (RA) increased the differentiation rate to 12.73% ± 1.77%, and upregulated meiotic markers, the resulting OLCs displayed abnormal epigenetic profiles (significantly lower 5mC, H3K4me3, and H3K9me3, but elevated 5hmC) compared with normal porcine GV oocytes (Wang et al., 2023). Critically, across all porcine studies to date, no functional oocytes capable of fertilization or embryonic development have been obtained. These collective findings suggest the potential presence of FGSC-like cells in pigs across developmental stages, but lack definitive functional validation of their stem cell identity or physiological relevance.

It is worth noting that the most robust functional validation of FGSCs to date comes from mice rather than livestock. For instance, FGSCs isolated from neonatal mouse ovaries successfully differentiated into functional oocytes and yielded viable offspring upon transplantation into chemotherapy-sterilized recipients, providing definitive evidence for their functional potential (Zou et al., 2009). However, the physiological significance of FGSCs remains debated even in mice; lineage-tracing experiments have failed to detect any contribution of putative stem cells to the oocyte pool under normal physiological conditions (Zhang et al., 2014). This suggests that if FGSCs exist, they may not measurably contribute to oocyte renewal *in vivo* without experimental manipulation.

At present, the field has not reached a consensus on the existence and functional significance of FGSCs in pigs or other mammals. Key challenges that must be overcome include: (i) the development of more specific and reliable markers to unequivocally distinguish prospective FGSCs from somatic cells and differentiated germ cells; (ii) the establishment of standardized, reproducible culture and differentiation protocols; and (iii) definitive *in vivo* functional validation through transplantation assays, the gold standard demonstrated in mice (Zou et al., 2009) but not yet achieved for any livestock species. Until such evidence is available, the biological and translational significance of pFGSCs remains uncertain.

## 6 Other adult stem cells

Pigs are a valuable source of diverse adult stem cells with potential applications in agricultural research and biotechnology. These include adipose-derived stem cells (Arrizabalaga and Nollert, 2017), muscle stem cells, porcine mesenchymal stem cells derived from bone marrow and umbilical cord (Huang et al., 2015), pancreatic stem cells (Han et al., 2015), dental germ stem cells (Gurel Pekozer et al., 2018), adult porcine skin-derived stem cell-like cells (Lermen et al., 2010), and intestinal stem cells (Stieler Stewart et al., 2018). Each cell type contributes distinctly to both fundamental biological research and practical agricultural applications.

Porcine adipose-derived stem cells (pASCs) exhibit a fibroblast-like morphology and have a cell cycle lasting 60–80 h (Williams et al., 2008; Wang et al., 2008; Schwarz et al., 2012). These cells can undergo 30–40 population doublings without entering replicative senescence (Williams et al., 2008; Wang et al., 2008). The immunophenotype of pASCs includes mesenchymal stem cell markers such as CD29, CD44, CD90, and CD105 (Chen et al., 2016; Dariolli et al., 2013; Brückner et al., 2014; Hanson et al., 2016; Zhang et al., 2014b), while lacking hematopoietic and endothelial cell markers like CD14, CD45, and CD31 (Chen et al., 2016; Dariolli et al., 2013; Zhang et al., 2014b). This marker profile is essential for confirming the purity of the cell population. pASCs are capable of differentiation into multiple lineages, including adipogenic, osteogenic, and chondrogenic lineages, and have been successfully induced into hepatocytes, neurons, and pancreatic islet-like clusters (Casado et al., 2012; Arrigoni et al., 2009; Bionaz et al., 2015; Brückner et al., 2014; Stock et al., 2010; Huang et al., 2007; Liu et al., 2017). Furthermore, pASCs can be reprogrammed into iPSCs (Zhang, et al., 2014b; Tang et al., 2012). Their differentiation potential is comparable to that of other porcine adult stem cells derived from bone marrow, peripheral blood, synovial membrane, and skin (Rho et al., 2009; Ock et al., 2016; Bharti et al., 2016).

Porcine skeletal muscle stem cells (PMSCs), also known as satellite cells, are vital for muscle maintenance and regeneration through their ability to proliferate, differentiate, and fuse with existing muscle fibers (Yin et al., 2013; Tierney and Sacco, 2016). These cells are gaining significant attention in the context of cultured meat production, as they can form muscle fibers *in vitro* (Li et al., 2022; Zhu et al., 2022).

A major challenge in their use, however, is the loss of stemness during prolonged *in vitro* culture, prompting research into pharmacological methods to preserve these properties (Ding et al., 2017). Pharmacological modulation of the Wnt/β-catenin pathway, specifically through GSK3β inhibitors like CHIR99021 and ASPP049, effectively promotes PMSC proliferation, prevents apoptosis, and maintains stemness markers such as Pax7 (von Maltzahn et al., 2012; Agley et al., 2017; Shi et al., 2022; Jones et al., 2015; Zammit et al., 2006; Park et al., 2022). While these advancements suggest the potential of PMSCs in cultured meat production, the risks of long-term GSK3β inhibition, including hyperproliferation and genomic instability, need careful consideration within both safety and ethical frameworks (Coller, 2019; Mademtzoglou and Relaix, 2022). Furthermore, translating these findings into viable agricultural applications faces major technical and economic hurdles. Key limitations include the scalability of bioprocesses, the high cost of culture media, and the reproducibility of producing stem-cell-derived tissues at a commercial scale. Ongoing research into molecular interactions and transcriptional regulation will be critical for optimizing the use of PMSCs and overcoming these translational challenges in agricultural applications.

## 7 Applications in agriculture

Collectively, these advancements in fundamental porcine stem cell biology have established a foundational technological platform for translational applications in agriculture. Fundamental breakthroughs now directly feed into applied cellular agriculture (cultured meat) and precision genetic breeding, while being continuously guided by ethical and regulatory considerations. This is particularly evident in two promising realms: cultured meat production and precision genetic breeding.

### 7.1 Cell sources, scaffolds, and culture systems for cultured meat

Cultured meat, produced through cellular agriculture, presents a potential complementary solution to the environmental, animal welfare, and food safety concerns associated with conventional livestock farming (Tuomisto and de Mattos, 2011; Pali-Schöll et al., 2019; Coleman et al., 2022). While life cycle assessments project significant reductions in land, water, and greenhouse gas emissions, industrial-scale realization faces critical bottlenecks: the limited proliferative capacity of primary cells, the high cost of growth media, challenges in replicating meat’s complex sensory qualities, and stringent regulatory frameworks. The production process essentially mimics natural myogenesis, in which muscle stem cells are expanded in bioreactors, often with scaffolds, and differentiated into muscle fibers (Ostrovidov et al., 2014; Piantino et al., 2024).

#### 7.1.1 Starting cell types and the challenge of scalability

**FIGURE 3:**
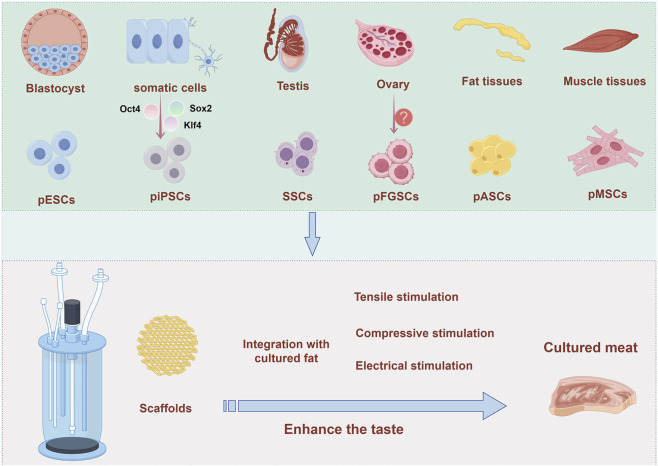
Applications of porcine stem cells in the production of *in vitro* cultured meat. Multiple cell types are utilized in the production of *in vitro* cultured meat, including porcine embryonic stem cells (pESCs), induced pluripotent stem cells (piPSCs), spermatogonial stem cells (SSCs)/female germline stem cells (pFGSCs) (though studies remain limited and controversial), adipose-tissue-isolated porcine adipose-derived stem cells (pASCs), and muscle tissue-isolated porcine muscle-derived stem cells (pMSCs). Cultured meat is produced by culturing these source cells on an appropriate scaffold. In addition to incorporating fat cells, various biophysical stimuli can be applied during the culture process, such as tensile stimulation, compressive stimulation, and electrical stimulation. These interventions have the potential to enhance the taste of cultured meat.

The selection of high-viability and safe seed cells is decisive (Figure 3). Tissue-derived satellite cells (muscle stem cells, MuSCs) are the most widely studied due to their strong myogenic potential (Yoshida et al., 2025). However, their differentiation capacity declines during extended culture, they are subject to the Hayflick limit, and maintaining myogenic identity during expansion remains difficult (Redondo et al., 2017). Recent single-cell RNA sequencing has revealed that MuSC fate divergence is highly dependent on seeding density: high-density culture preserves myogenic potential, whereas low-density conditions promote undesirable transition into fibro/adipogenic progenitors (FAPs), highlighting a critical parameter for optimizing large-scale procedures (Zhang et al., 2026).

Pluripotent stem cells (ESCs and iPSCs) and immortalized cell lines offer indefinite proliferation, circumventing senescence barriers (Genovese et al., 2017; Pasitka et al., 2023). Porcine pre-gastrulation epiblast stem cells (pgEpiSCs) exhibit stable clonal proliferation and genomic stability (Zhu, et al., 2023a). A recent breakthrough leveraged stable porcine pgEpiSCs in a completely serum- and animal-component-free differentiation system to achieve multidirectional differentiation into muscle and adipose tissues, generating multi-tissue cultivated meat that better replicates the complex composition of conventional meat (Yao et al., 2026). While immortalization can be achieved through genetic engineering (e.g., CRISPR/Cas9-mediated knockout of TP53 or overexpression of hTERT), such approaches carry potential risks of exogenous gene integration, genomic instability, and tumorigenicity, raising substantial regulatory and consumer acceptance hurdles. In contrast, spontaneous immortalization, as demonstrated by Pasitka et al. (2023), can yield cell lines with robust genetic stability and no tumorigenic potential, thereby offering a safer route to address these safety and regulatory concerns (Pasitka et al., 2023). The field thus faces a clear trade-off: primary cells have a simpler safety profile but limited scalability, while pluripotent and immortalized lines offer scale at the cost of extensive safety and public acceptance testing, though strategies like spontaneous immortalization may significantly lower this barrier. Future research must balance scalability, safety, and regulatory compliance to identify the most commercially viable cell source.

#### 7.1.2 Scaffolds, fat integration, and meat quality replication

Replicating the texture, flavor, and mouthfeel of conventional meat is paramount for consumer acceptance, requiring advanced scaffold designs and the integration of cultured fat. Scaffolds provide physical support for cell attachment and differentiation by mimicking the native extracellular matrix (ECM) environment (Specht et al., 2018). While animal-derived materials, such as collagen and gelatin, are effective (MacQueen et al., 2019), they contradict the sustainability aims of cultured meat. Consequently, plant-derived materials (e.g., soy protein, zein, peanut protein, sorghum prolamin) and textile-derived scaffolds have gained prominence for their low cost, edibility, and demonstrated cytocompatibility (Zheng et al., 2022a; Wang et al., 2025b; Kim et al., 2024a).

Various processing techniques, such as freeze-drying, electrospinning, and 3D bioprinting, have been employed to tailor these materials into adaptive bio-orchestrating anisotropic scaffolds (ABS) that mimic traditional meat textures (Lv and Feng, 2006; Zhang et al., 2005; Jung et al., 2025b). However, critical bottlenecks hinder industrial-scale implementation. Production scalability challenges arise from the high energy and time costs of techniques like electrospinning and 3D bioprinting at commercial volumes, alongside batch-to-batch variability in scaffold architecture (Gurel et al., 2024). Furthermore, bulk procurement of plant-based feedstocks risks disrupting existing food supply chains, and novel biomaterials face strict food-grade regulatory approval requirements.

The integration of adipose tissue is equally critical for flavor development. Adipose-derived stem cells (ADSCs) can be differentiated into adipocytes on 3D microcarriers or within scaffolds to replicate intramuscular fat (Song et al., 2022), and optimizing differentiation conditions (e.g., specific combinations of dexamethasone, insulin, and rosiglitazone) can yield a favorable fatty acid profile, such as a beneficial ω-6/ω-3 ratio (Liu et al., 2024). To further enhance texture, advanced scaffolds like chitin-sodium alginate-quercetin (CH-SA-QR) have been shown to support myotube fusion, yielding tenderness comparable to traditional beef (Wang et al., 2025b). Physical interventions, including cyclic mechanical stretching, electrical stimulation, and specific ECM proteins like laminin, significantly improve collagen organization, parallel myotube formation, and overall structural maturation (Kim et al., 2024b; Troop and Puetzer, 2024). Despite these advances, scaling these maturation processes poses significant economic challenges. The high cost of recombinant proteins, energy-intensive mechanical/electrical stimulation protocols, and the complexity of food-grade certification for novel scaffolds must be addressed for commercial viability.

#### 7.1.3 Optimization of serum-free culture systems

The transition from fetal bovine serum (FBS) to chemically defined, serum-free media (SFM) is imperative to reduce cost, compositional variability, and ethical concerns (Dai et al., 2024; Kolkmann et al., 2020). Systematic optimization has shifted from simple basal media to sophisticated formulations tailored to muscle cell biology. Notable progress includes the use of specific supplements like naringenin (Guan et al., 2023) or recombinant porcine FGF1 (Liu et al., 2025) to promote differentiation and expansion, as well as cost-effective substitutes such as *Vibrio* natriegens lysate-based media (VN40) (Dolgin et al., 2025) and plant-derived oilseed protein isolates (Beefy-9) (Stout et al., 2022; Stout et al., 2023). Microfluidic platforms have further enabled precise microenvironmental control (Zhang et al., 2025).

A deeper understanding of cellular metabolism during myogenesis is now driving a new wave of optimization. Dynamic shifts occur from glycolysis during proliferation to oxidative phosphorylation during differentiation, suggesting that stage-specific media formulations can improve both efficiency and product quality (Yeon Jung et al., 2024; Jung D. Y. et al., 2025). Furthermore, non-destructive monitoring of metabolite biomarkers (e.g., γ-glutamyl-L-leucine) in spent media may serve as a quality control strategy to detect suboptimal cell states during large-scale culture (Yeon Jung et al., 2024). Despite these advances, persistent translational bottlenecks remain: the reliance on expensive recombinant growth factors limits cost-effectiveness, species-specificity of many SFM formulations complicates multi-species platforms, and the introduction of novel components (e.g., bacterial lysates) requires extensive safety validation under current food regulatory frameworks.

In summary, cultured meat production is advancing through coordinated innovations in seed cell sourcing, scaffold engineering, and serum-free culture systems. Key enabling strategies include the isolation of high-purity MuSCs by simple, low-cost methods such as ice-cold treatment (Huang et al., 2025), modulation of signaling pathways (e.g., Notch, Wnt) to enhance MuSC proliferation and stemness (Qin et al., 2025; Suriya et al., 2024), the generation of multi-tissue cultivated meat from pluripotent stem cells (Yao et al., 2026), and the development of edible, plant-based scaffolds that support both muscle and fat maturation. However, achieving commercial viability demands that the integrated challenges of scalability, cost-efficiency (particularly for growth factors and complex scaffolds), regulatory approval for novel materials and immortalized cell lines, and sensory profiles that meet consumer expectations be overcome. Future progress will depend on a holistic systems approach that tightly integrates bioprocess engineering, material science, and safety assessments to move cultured meat from laboratory promise to industrial reality.

### 7.2 Stem cell-based breeding

**FIGURE 4:**
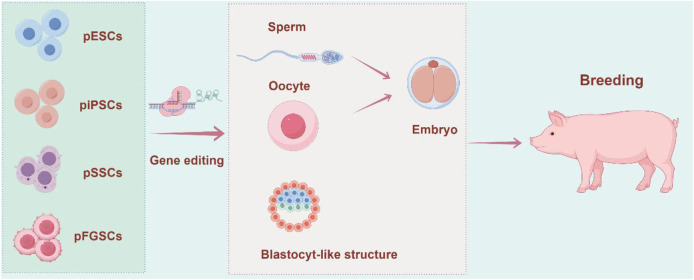
Applications of porcine stem cells in obtaining new breeding materials. Porcine embryonic stem cells (pESCs), porcine induced pluripotent stem cells (piPSCs), and spermatogonial stem cells (SSCs)/female germline stem cells (pFGSCs) can differentiate into sperm or oocyte, or directly into blastocyst-like structures. Gene editing can be done as per new variety breeding requirements during this, making the resultant sperm, oocyte, and blastocyst-like structures have the desired genotypes. *In vitro* differentiated sperm and oocytes can be *in vitro* fertilized to get embryos, which, like blastocyst-like structures, can be used to produce live pigs with the desired genotypes, thus obtaining new breeding materials.

Stem cell-based breeding represents a novel approach to enhancing and optimizing livestock breeds, capitalizing on the intrinsic properties of stem cells, namely, their capacity for self-renewal and differentiation into a myriad of cell types. A conceptual framework for this approach, termed *in vitro* breeding, has been proposed by Goszczynski et al. (2019). This strategy combines genomic selection, a widely used approach that estimates the genetic merit of individuals based on genome-wide DNA markers without requiring phenotypic measurements, with ESC derivation and *in vitro* germ cell differentiation. In this scheme, embryos are generated *in vitro* from high-genetic merit parents; ESC lines are derived from each embryo and genotyped to calculate estimated embryonic breeding values; only cell lines with the highest genetic merit are then advanced to *in vitro* gametogenesis, followed by a new round of *in vitro* fertilization, selection, and germ cell generation. A simulation comparing this approach to conventional genomic selection in Holstein cattle projected that *in vitro* breeding could complete approximately 100 generations in 25 years, compared to only 10 generations under genomic selection alone, thereby dramatically accelerating genetic gain (Goszczynski et al., 2019). Although the realization of this strategy depends on the availability of robust germ cell differentiation protocols in livestock species, which remain under development, it illustrates how stem cell technologies could fundamentally shorten breeding timelines (Figure 4).

Importantly, the reconstitution of germ cells from PSCs marks a significant breakthrough that has already been validated in rodent models. Foundational studies identified critical factors for primordial germ cell (PGC) specification (Ohinata et al., 2009), paving the way for the production of functional gametes and viable offspring. For example, mouse ESCs/iPSCs can differentiate into PGC-like cells (PGCLCs) which, when aggregated with embryonic gonadal somatic cells, mature into functional spermatids or oocytes capable of producing healthy pups (Hayashi et al., 2012; Zhou et al., 2016)*.* Subsequent refinements have reconstituted oogenesis entirely *in vitro* (Hikabe et al., 2016) and improved the overall efficiency of PSC-to-germ-cell protocols (Yoshino et al., 2021).

These proof-of-principle studies have since been extended beyond mice. Functional gametes capable of contributing to offspring have been successfully generated from rat PSC-derived germ cells (Oikawa et al., 2022). In human cells, progress has likewise been notable at stages prior to complete gametogenesis: recent studies achieved the generation of abundant mitotic pro-spermatogonia and oogonia-like cells from human PSCs (Murase et al., 2024). While the production of fully functional human gametes remains subject to substantial technical and ethical constraints, these models provide valuable materials for studying germline development.

For larger domestic species such as pigs, pluripotent stem cell technology has begun to show promise but has not yet produced the same level of functional *in vivo* validation as in rodents. piPSCs and pEPSCs have been shown to express key germ cell markers and to differentiate toward germ cell lineages under defined conditions (Wang et al., 2016; Gao et al., 2019; Wang et al., 2015). These efforts have drawn on protocols originally developed in mice, in which PSCs are first differentiated into epiblast-like cells and subsequently into PGCLCs via stimulation with BMP4, LIF, SCF, and EGF (Hayashi et al., 2011; Wang et al., 2016). However, critical species-specific differences exist: for instance, porcine PGCs appear to originate from the posterior pre-primitive-streak competent epiblast through sequential upregulation of SOX17 and BLIMP1 in response to WNT and BMP signaling, with weak and apparently cytoplasmic expression of PRDM14, a pattern that departs from the mouse paradigm (Kobayashi et al., 2017). These differences highlight the need to tailor *in vitro* germ cell induction protocols to porcine-specific developmental programs, rather than simply adapting mouse conditions. Parallel advances in generating embryo-like structures provide complementary *in vitro* platforms. Blastoids derived from PSCs mimic pre-implantation blastocysts and have been produced from mouse and human PSCs under specific 3D culture conditions (Rivron et al., 2018; Li et al., 2019; Yu et al., 2021; Liu, et al., 2023a). These structures contain analogs of the trophoblast, epiblast, and hypoblast lineages, thereby recapitulating key features of early embryos without requiring the use of fertilized oocytes. Recent work reports porcine blastoids generated from stable pESCs using a 3D two-step differentiation strategy that resemble natural blastocysts in morphology, size, and cellular composition (Xiang et al., 2024). These porcine blastoids can survive and expand *in vitro* for extended periods (Xiang et al., 2024), offering tractable models for early embryogenesis and for testing PSC-based breeding interventions.

Taken together, these staged successes underscore the potential of PSC-based breeding to increase genetic gain and shorten breeding cycles, while also highlighting the translational barriers that must be addressed for application in livestock. Key challenges include: (i) the lack of robust, species-optimized protocols for complete *in vitro* gametogenesis in pigs and cattle; (ii) the long-term genetic and epigenetic stability of PSC lines during extended culture and *in vitro* gametogenesis; (iii) the need to prevent losses in genetic variance when applying intense *in vitro* selection; and (iv) ethical and regulatory frameworks that have yet to be established for food products derived from stem cell-based breeding.

## 8 Summary and outlook

Research on porcine stem cells, including pESCs, piPSCs, pGSCs (spermatogonial stem cells and female germline stem cells), and adult stem cells, has advanced substantially, establishing a foundational platform for agricultural biotechnology. While significant progress has been made in isolation, characterization, and *in vitro* manipulation, critical challenges persist, defining the immediate and future research agenda. Notably, the field currently lacks stable, germline-competent pluripotent stem cell lines that can be reproducibly derived and maintained across laboratories, which represents a fundamental barrier to translational applications in breeding.

For pESCs, the foremost challenge is establishing cell lines with robust germline transmission capability, essential for precision breeding applications. Multiple laboratories have reported porcine ESC-like lines, yet few, if any, have demonstrated unequivocal germline competence, and protocols successful in one laboratory often prove difficult to reproduce elsewhere. This variability underscores a deeper conceptual gap: the pluripotent state in pigs remains insufficiently characterized compared to mouse and human counterparts. A critical examination reveals that porcine ESCs struggle with germline competence largely due to profound epigenetic barriers, such as aberrant DNA methylation or genomic imprinting errors during prolonged culture, and culture condition mismatches that fail to capture the true ground state of pluripotency (Wang et al., 2025; Shi et al., 2025). Future efforts should prioritize elucidating the unique signaling and epigenetic networks governing porcine pluripotency. Important guidance can be drawn from comparative studies of naïve versus primed pluripotency in rodents and primates; however, naïve pluripotency, characterized by domed colony morphology, LIF-dependency, and robust chimera contribution in mice, has proven difficult to establish and stabilize in ungulate species, likely reflecting species-specific differences in early embryonic development and pluripotency regulation (Goszczynski et al., 2019). Leveraging single-cell multi-omics technologies will be crucial to map cellular heterogeneity and identify the precise conditions that sustain a developmentally competent, naïve-like state *in vitro*.

A central lesson from comparative pluripotency research is that the pig is neither a scaled-up mouse nor a miniature human. Porcine pluripotent cells exhibit unique signaling dependencies (e.g., predominant FGF/ERK activity, limited LIF/STAT3 response), distinct chromatin landscapes (delayed X-chromosome reactivation, aberrant imprinting patterns), and a recalcitrance to naïve state capture that is not observed in rodents. Recognizing this species specificity is not merely academic; it is a prerequisite for the rational design of culture media, reprogramming strategies, and differentiation protocols that work reliably in pigs. Future breakthroughs will depend on deconstructing the porcine pluripotency network on its own terms, rather than forcing it into mouse or human frameworks.

The generation of piPSCs provides a vital alternative, yet their widespread application is hampered by the low efficiency and genomic instability of current reprogramming methodologies. The field must move toward standardized, integration-free reprogramming protocols. A key focus should be on understanding and overcoming the barriers to complete endogenous pluripotency network activation, potentially through the use of novel small-molecule cocktails or species-specific reprogramming factors to improve safety and consistency (Zhou et al., 2023). Furthermore, variability in the differentiation propensity of piPSC lines, where some clones retain epigenetic memory of their somatic origin, remains an underappreciated challenge that complicates both basic research and translational use.

In the realm of pGSCs, the potential for revolutionizing animal breeding is immense. The major bottleneck for SSCs is the lack of definitive species-specific molecular markers, hindering high-purity isolation and functional studies. Regarding FGSCs in postnatal pig ovaries, it is critical to note that their existence and functional relevance remain a matter of considerable scientific debate. While several studies have reported the isolation and characterization of putative FGSCs, other evidence suggests that neo-oogenesis does not occur naturally in adult mammalian ovaries, and that cells interpreted as FGSCs may represent *in vitro* culture artifacts or misidentified ovarian cell populations. At present, the field has not reached consensus on this issue. Therefore, further rigorous validation, including lineage-tracing experiments and functional transplantation assays with robust controls, is required before FGSCs can be positioned as a reliable platform for breeding applications.

Adult stem cells, such as skeletal muscle stem cells (PMSCs) and adipose-derived stem cells (pASCs), are directly applicable to cultivated meat production. The primary challenge here is the loss of stemness and differentiation potential during large-scale expansion. Addressing this requires the development of advanced, chemically defined culture media and bioreactor systems that mimic the native niche, ensuring consistent cell quality for industrial-scale applications. A related concern is the absence of standardized quality-control metrics for stemness and differentiation capacity in large-scale cell banks, which is essential for regulatory approval and batch-to-batch reproducibility in food production contexts.

Looking forward, the translational success of porcine stem cell research hinges on pivotal progress within three deeply interconnected domains: fundamental biology, scalable bioprocessing, and regulatory preparedness. First, a deeper understanding of porcine-specific pluripotency states and germ cell specification programs, building on comparative insights from mouse, human, and other large animal models, is needed to establish stable, germline competent cell lines that can be reproducibly generated across laboratories. Second, for cultivated meat applications, research-scale differentiation protocols must be translated into cost competitive industrial bioprocesses; this requires not only serum-free media and edible scaffolds but also rigorous quality control frameworks that ensure product consistency and safety. Third, the development of proactive ethical and regulatory guidelines must proceed in parallel with scientific advances. Critical issues, including the regulatory status of stem cell-derived food products, the management of genetic variance in *vitro* breeding schemes, and the alignment of animal welfare standards with novel production systems, require early and sustained engagement with policymakers and the public to enable responsible innovation. Progress in any one of these areas alone will be insufficient; only through their synergistic advancement can the potential of porcine stem cells be realized in contributing to sustainable agriculture and global food security.
