# Supplementary figures and images for: Advances in porcine stem cell research and their applications in agriculture
Source: Front Cell Dev Biol. 2026 Jun 29;14:1862107. doi: 10.3389/fcell.2026.1862107 (PMC13357440; doi:10.3389/fcell.2026.1862107)

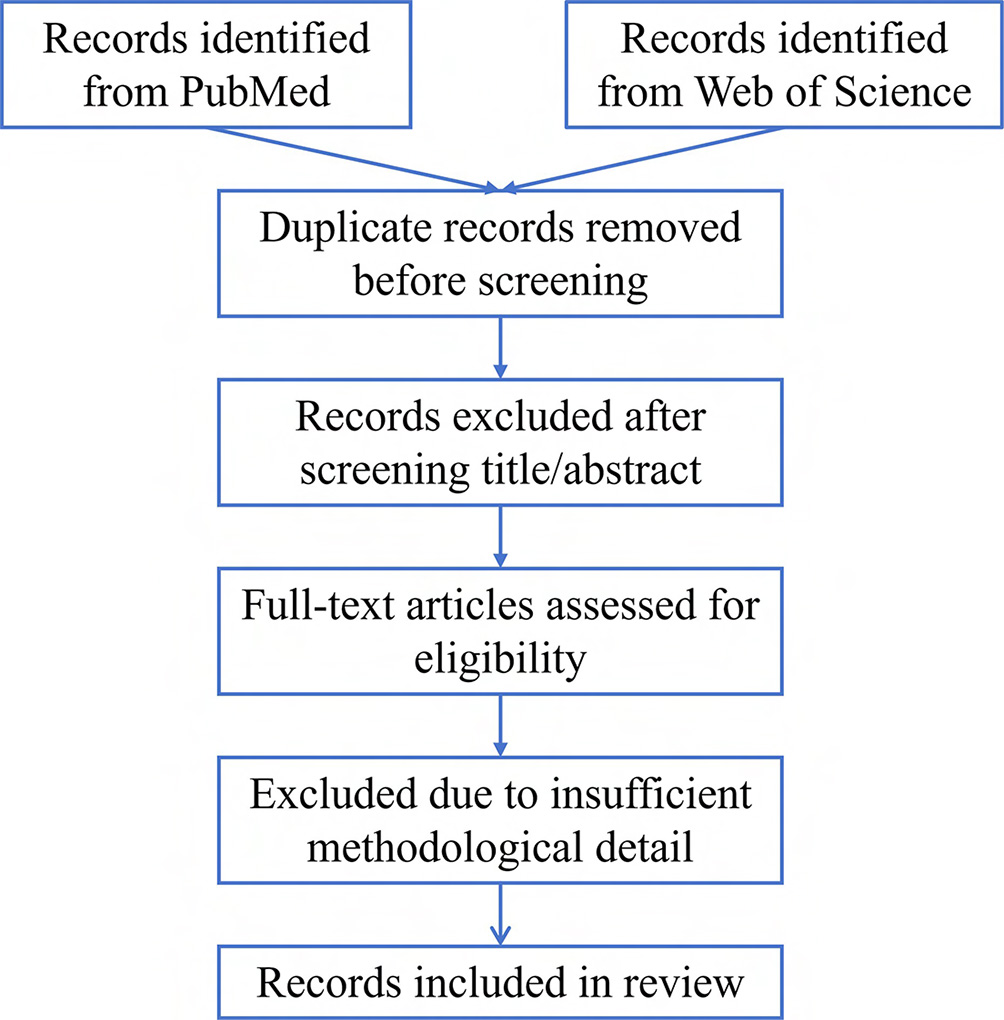

Supplement: Supplementary file 2 [file Image1.jpg]
